# Comorbidities and chance of remission in patients with early rheumatoid arthritis receiving methotrexate as first-line therapy: a Swedish observational nationwide study

**DOI:** 10.1136/rmdopen-2023-003714

**Published:** 2023-12-20

**Authors:** Liselotte Tidblad, Helga Westerlind, Bénédicte Delcoigne, Johan Askling, Saedis Saevarsdottir

**Affiliations:** 1 Clinical Epidemiology Division, Department of Medicine, Solna, Karolinska Institutet, Stockholm, Sweden; 2 Faculty of Medicine, School of Health Sciences, University of Iceland, Reykjavik, Iceland

**Keywords:** Arthritis, Rheumatoid, Methotrexate, Epidemiology, Outcome Assessment, Health Care

## Abstract

**Objectives:**

This study aims to examine whether comorbidities affect the likelihood of reaching primary remission on methotrexate monotherapy as the first disease-modifying antirheumatic drug (DMARD) in early rheumatoid arthritis (RA).

**Methods:**

We used nationwide Swedish clinical and quality registers to collect RA disease activity measures and comorbidity data for patients diagnosed with RA 2007–2020 (n=11 001). The primary outcome was failure to reach 28-joint Disease Activity Score (DAS28) remission at 3 months. Secondary outcomes included Boolean, Simplified Disease Activity Index/Clinical Disease Activity Index remission, European Alliance of Associations for Rheumatology response and no swollen joint count at 3 and 6 months. For each comorbidity, and for combinations thereof, we calculated adjusted relative risks (RRs) of failure to reach remission, using modified Poisson regression.

**Results:**

In total, 53% (n=4019/7643) failed to reach DAS28 remission after 3 months of methotrexate monotherapy, ranging from 66% (n=25/38) among patients with chronic kidney disease to 48% (n=154/319) in patients with previous cancer. The risk of not reaching DAS28 remission at 3 months (RR adjusted for sex and age) was increased among patients with endocrine (RR 1.08, 95% CI 1.01 to 1.15), gastrointestinal (RR 1.16, 95% CI 1.03 to 1.30), infectious (RR 1.21, 95% CI 1.06 to 1.38), psychiatric (RR 1.24, 95% CI 1.15 to 1.35) and respiratory comorbidities (RR 1.16, 95% CI 1.01 to 1.32). Having three or more comorbidity categories was associated with a 27% higher risk of DAS28 remission failure at 3 months. A similar pattern was observed for the secondary outcomes.

**Conclusions:**

Comorbidities decrease the chance of reaching remission on methotrexate as DMARD monotherapy in patients with early RA and are important to consider when assessing treatment outcomes.

**WHAT IS ALREADY KNOWN ON THIS TOPIC**

- Several comorbidities have been associated with a higher disease activity and lower remission rate in rheumatoid arthritis (RA). However, most previous studies on comorbidities in RA have been conducted in patients with established disease.

**WHAT THIS STUDY ADDS**

- In order to examine the effect of comorbidities on remission in early RA, only patients with early RA, treated with methotrexate in monotherapy as the first disease-modifying antirheumatic drug, were included in this study. Patients with psychiatric, infectious, respiratory, gastrointestinal and endocrine comorbidities had an increased risk of remission failure. The strongest associations were observed for patients with psychiatric comorbidities. Higher overall comorbidity burden was also associated with failure to reach remission.

**HOW THIS STUDY MIGHT AFFECT RESEARCH, PRACTICE OR POLICY**

- Comorbidities associated with the likelihood of remission in early patients with RA, treated with methotrexate monotherapy and are important to consider when assessing treatment outcomes.

## Introduction

Comorbid conditions affect patients with rheumatoid arthritis (RA) in several ways. First, comorbidities are considered important for the excess mortality seen in patients with established RA, as some comorbidities develop at a higher rate in patients with RA, due to shared risk factors and direct and indirect effects of chronic inflammation.^1–4^ We have previously found differences in the prevalence of certain comorbidities already at RA diagnosis, with a higher prevalence of diabetes, respiratory and thyroid comorbidities and a lower prevalence of malignancies and psychiatric disorders compared with the general population.^5^

Second, comorbidities can affect the choice of antirheumatic treatment. For example, methotrexate (MTX), the first-line treatment in RA,^6 7^ is cautioned in patients with chronic kidney disease (CKD). For instance, we have demonstrated that MTX is the most commonly used disease-modifying antirheumatic drug (DMARD) in early RA overall, but as expected with a lower use in patients with CKD and respiratory comorbidities.^8^ Third, antirheumatic treatment can lead to side effects and the development of additional comorbidities, such as haematological and hepatic toxicity, as well as infections.^9 10^

With regard to the clinically relevant question of how comorbidities affect RA disease activity and response to treatment including chances of reaching remission, results so far are conflicting. In established RA, the presence of cardiovascular diseases, diabetes and depression have been associated with higher RA disease activity and lower response to treatment.^11–13^ Also, the cumulative burden of comorbidities has been associated with inferior treatment response in established disease.^14–16^ By contrast, other studies have reported that comorbidities neither influence RA disease activity nor remission rates.^17 18^

Most, though not all, of these previous studies on comorbidities and RA remission have examined patients with established RA, or included patients on different antirheumatic treatments.^11 13 14 16–18^ To shed more light on this issue, the aims of this study were, therefore, to examine if specific comorbidities and the overall comorbidity burden are associated with the failure to reach primary remission, focusing on early RA and on one defined RA treatment (here: MTX monotherapy as the first DMARD).

## Methods

### Data sources

In this study, we linked nationwide Swedish registers, described in online supplemental table S1, using the personal identity number unique to residents in Sweden as linkage key. In short, the Swedish Rheumatology Quality Register (SRQ), covering over 85% of all patients with RA in Sweden,^19^ was used to identify patients with incident RA. SRQ is used at RA diagnosis and at follow-up visits, as a part of the clinical routine care. SRQ was used for information on symptom duration, DMARD use and serological status as well as for clinical information on disease activity to calculate outcome measures.

10.1136/rmdopen-2023-003714.supp1 Supplementary data

Information on comorbidities was captured through the National Patient Register (NPR), where International Classification of Diseases (10th revision, ICD-10) diagnoses from hospital discharge and non-primary outpatient care visits are registered and from the Swedish Cancer Register. Information about prescribed and dispensed drugs was captured from the Prescribed Drug Register (PDR). The Education Register was used to retrieve data on the highest attained educational level.

### Study population

The data extraction and cohort have previously been described.^20^ In brief, patients from SRQ were included if they were 18 years of age or older, registered in SRQ with the diagnosis of RA and prescribed MTX between 1 January 2007 and 31 October 2020. The index date for RA diagnosis was counted as the start date of MTX in SRQ.

**Figure 1:**
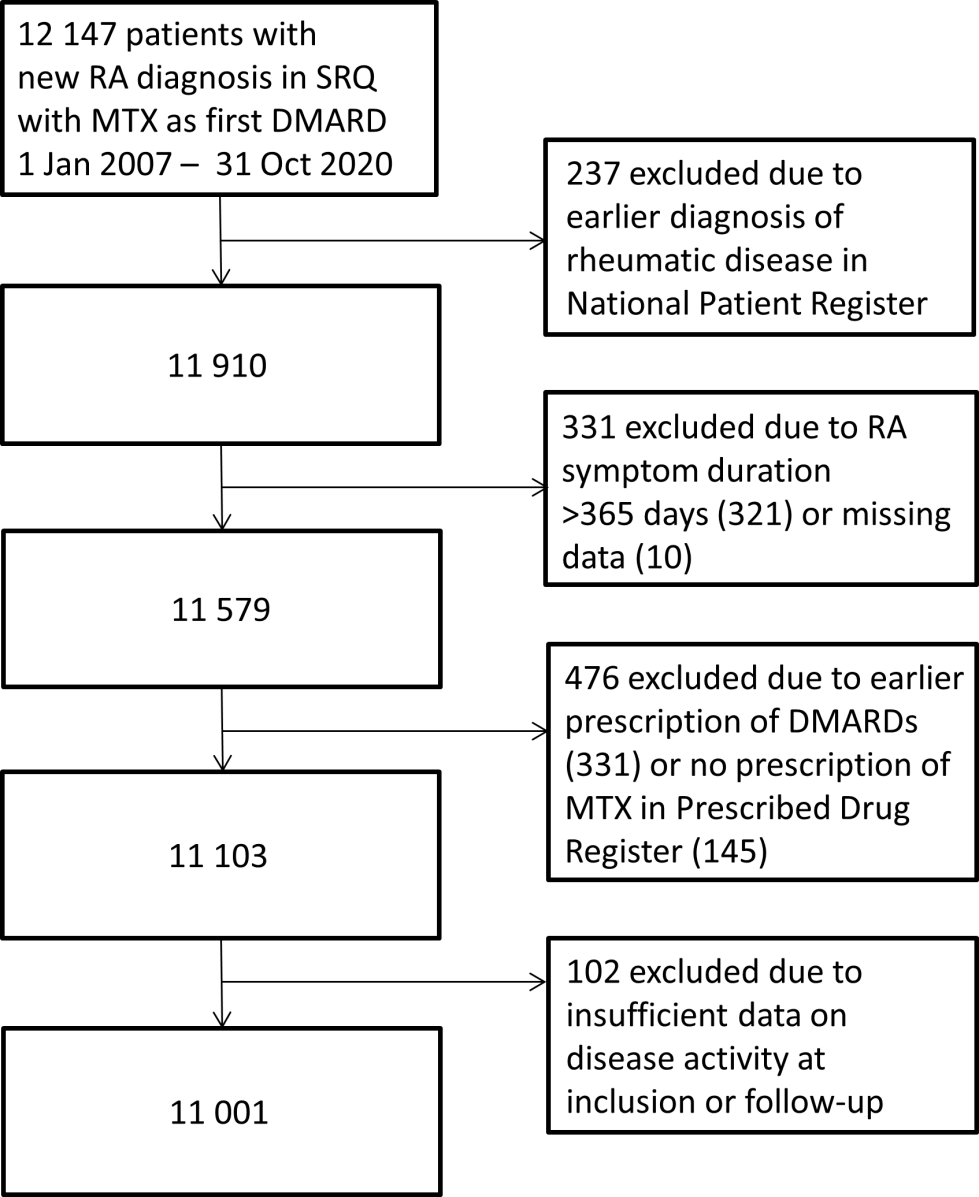
Flow chart showing selection of the study population. DMARD, disease-modifying antirheumatic drug; MTX, methotrexate; RA, rheumatoid arthritis; SRQ, Swedish Rheumatology Quality register.

MTX had to have been prescribed as first ever DMARD, and dispensed according to PDR, in a window from 1 month before to 1 month after the index date. To avoid prevalent RA, patients were excluded if diagnosis codes for a rheumatic disease had been registered in the NPR more than 1 year before the index date, if the reported symptom duration in SRQ was longer than 1 year, or if there were registered dispensations of DMARDs according to the PDR within 18 months up until the initiation of MTX (see figure 1) (online supplemental table S2 for excluded diagnoses and drugs). Furthermore, there had to be data on at least one visit registered in SRQ during the follow-up period (ie, during an interval from 90 days before to 269 days after the MTX start date).

To identify a 3-month and 6-month return visit, we searched for visits in the window of 31–149 days for the 3-month visit, and 150–269 days for the 6-month visit. If there were several follow-up visits during this time period, we used the visit/s with: (1) the highest completeness in outcome components, (2) closest in time to 90 and 180 days, respectively and (3) in case MTX was discontinued, the last visit that occurred during treatment with MTX was used. Information about how the selection of visits and assessment of treatment outcomes were handled has been described in detail elsewhere.^20^

Patients were regarded as seropositive if the ICD 10-code registered in SRQ as rheumatoid factor (RF)-citrullinated and/or anti-citrullinated protein antibodies (ACPA)-positive (M05, M06.0L, M06.8L), and as seronegative if RF-negative and ACPA either negative or unspecified (M06.0, M06.0M, M06.0N, M06.8M, M06.8N).

### Comorbidity data

Comorbidity data were collected from 5 years prior to the index date and up until that date, and defined through ICD-10 codes from hospital discharge and non-primary outpatient care visits from NPR as well as from the Swedish Cancer Register. In addition, we used drug dispensations for antidepressants, oral antidiabetics, insulin, lipid-lowering drugs, thyroid, antithyroid and antidementia drugs from PDR from 18 months before and up until the index date to further expand the search for comorbidities often managed in primary healthcare and for which there are specific treatments.

We defined 31 individual comorbidities and grouped these into 10 comorbidity categories: cardiovascular, non-cardiac vascular, malignant, endocrine, gastrointestinal, infectious, chronic kidney, neurological, psychiatric and respiratory diseases, using the same approach as in our previous studies.^5 8^ For infectious diseases, we limited the ICD-10 codes to hospitalisations with an infectious disease as main diagnosis. Online supplemental table S3 presents the Anatomical Therapeutic Chemical Classification and ICD-10 codes used.

We used the Rheumatic Disease Comorbidity Index (RDCI) as a measure of disease burden. RDCI is a weighted index that covers lung diseases, cardiovascular diseases, hypertension, fractures, depression, diabetes, cancer and ulcer/stomach problems (online supplemental table S4).^21–23^ As an alternative measure of disease burden, we also assessed the sum of unique comorbidity categories by the time of RA diagnosis.

### Primary and secondary outcomes

The primary outcome was 28-joint Disease Activity Score (DAS28) remission (defined as DAS28<2.6 or DAS28-CRP<2.4). Secondary outcome measures comprised CDAI (Clinical Disease Activity Index) remission, SDAI (Simplified Disease Activity Index) remission, American College of Rheumatology/European Alliance of Associations for Rheumatology (ACR/EULAR) Boolean remission (with the original patient’s global assessment (PGA) threshold of 10 mm), EULAR good response compared with moderate/no response and no swollen joints (using 28 joints). Definitions of the remission outcomes are shown in online supplemental table S5^24–28^. The primary follow-up time point was at 3 months, and secondary follow-up was at 6 months following start of MTX in DMARD monotherapy. Information on disease activity (DAS28/DAS28-CRP, CDAI, SDAI and their individual components: 28-swollen and 28-tender joint counts (SJC/TJC), erythrocyte sedimentation rate (ESR), C reactive protein (CRP), the evaluator’s global assessment (EGA), PGA, pain on a Visual Analogue Scale (VAS) and functional impairment evaluated with Health Assessment Questionnaire (HAQ)) was collected at each follow-up visit.

### Statistical analysis

We calculated the disease activity (median/IQR for DAS28, DAS28-CRP, SJC, TJC, CRP, ESR, PGA, HAQ and VAS pain) at RA diagnosis, and the proportion that reached DAS28 remission at 3 and 6 months, within each comorbidity category.

We used modified Poisson regression to evaluate the association between our exposures (comorbidities) and primary and secondary outcome measures (remission), with all comorbidity categories incorporated in the same model: crude, adjusted for sex and age, and a full model adjusted for sex, age, serological status, smoking (never/ever), glucocorticoid treatment at MTX start (yes/no), educational level (≤9, 9–12 and >12 years) and calendar period (2007–2013, 2014–2020) and also with the comorbidity categories assessed separately, adjusted for sex and age.

We performed a separate analysis with modified Poisson regression where the comorbidity burden was assessed as RDCI and the number of comorbidity categories, respectively, adjusted for sex and age. We furthermore performed analyses stratified by sex and serological status, for the full model. Missing data were handled by using listwise deletion/complete case analysis. All statistical analyses were performed in SAS V.9.4.

## Results

### Patient characteristics

A total of 11 001 patients with MTX as first DMARD in monotherapy in new-onset RA were included (figure 1). Of these, 67% (n=7419) were female, and 69% (n=7560) were seropositive (table 1). The median age for all patients was 63 years (IQR 51–72); the seropositive patients 61 years (IQR 50–70) and the seronegative patients 67 years (IQR 56–74). Sixty per cent of the patients for whom we had data on smoking were ever smokers (n=5851).

**Table 1:** Baseline characteristics of patients treated with methotrexate in DMARD monotherapy at RA diagnosis

	All RA n=11 001	Seropositive RA* n=7560 (68.7%)	Seronegative RA* n=3147 (28.6%)
Female, n (%)	7419 (67.4)	5326 (70.4)	1901 (60.4)
Age, years, median (IQR)	62.8 (51.3–71.6)	61.2 (49.7–70.1)	66.5 (55.5–74.1)
Educational level†			
≤9 years, n (%)	2581 (27.6)	1690 (26.5)	823 (30.4)
10–12 years, n (%)	4327 (46.3)	2999 (47.0)	1197 (44.1)
>12 years, n (%)	2437 (26.1)	1695 (26.6)	692 (25.5)
Smoking‡, n (%)	5851 (59.8)	4200 (61.5)	1496 (55.1)

*Missing information on serological status: 294 (2.7%).

†Missing information on educational level: all RA cases: 1656 (15.1%), seropositive RA: 1176 (15.6%), seronegative RA: 435 (13.8%).

‡Current or previous smoking. Missing information on smoking: all RA cases: 1214 (11.0%), seropositive RA: 735 (9.7%), seronegative RA: 434 (13.8%).

DMARD, disease-modifying antirheumatic drug; RA, rheumatoid arthritis.

Overall, 56% of the included patients had a history of at least one of the defined comorbidity categories. Most comorbidity categories were slightly more frequent, and the comorbidity burden was higher, among the seronegative patients, who were also older (table 2). Males had a higher prevalence of cardiovascular and non-cardiac vascular comorbidities, whereas female patients had more psychiatric comorbidity. Males more often had an RDCI of ≥3 (14%) compared with the females (7%) (online supplemental table S6). The comorbidity prevalence increased with age for almost all of the comorbidity categories. For the age group 18–49 years, 36% had any of the comorbidity categories, compared with 59% among patients 50–74 years old and 75% of patients over the age of 75 (online supplemental table S7). All patients had at least one visit in SRQ; at RA diagnosis, 92% of the patients (n=10 088) had a registered visit in SRQ, 75% (n=8273) had a visit at 3 months, decreasing to 54% (n=5924) for the 6 months visit. The proportion that had a registered visit in the time window was similar for the different comorbidity categories (online supplemental table S8).

**Table 2:** Comorbidities in patients initiating methotrexate in DMARD monotherapy at RA diagnosis, overall and by serological status

	All RA at diagnosis n=11 001	Seropositive RA at diagnosis* n=7560	Seronegative RA at diagnosis* n=3147
Cardiovascular, n (%)	942 (8.6)	575 (7.6)	336 (10.7)
Non-cardiac vascular, n (%)	3163 (28.8)	1976 (26.1)	1093 (34.7)
Malignant, n (%)	467 (4.3)	283 (3.7)	167 (5.3)
Endocrine, n (%)	2234 (20.3)	1506 (19.9)	664 (21.1)
Gastrointestinal, n (%)	681 (6.2)	447 (5.9)	215 (6.8)
Infectious, n (%)	612 (5.6)	411 (5.4)	184 (5.9)
Chronic kidney disease, n (%)	61 (0.6)	37 (0.5)	23 (0.7)
Neurological, n (%)	770 (7.0)	495 (6.6)	258 (8.2)
Psychiatric, n (%)	1660 (15.1)	1118 (14.8)	497 (15.8)
Respiratory, n (%)	525 (4.8)	359 (4.8)	148 (4.7)
No comorbidity, n (%)	4804 (43.7)	3473 (45.9)	1216 (38.6)
RDCI: 0, n (%)	7554 (68.7)	5355 (70.8)	2006 (63.7)
RDCI: 1, n (%)	1602 (14.6)	1039 (13.7)	520 (16.5)
RDCI: 2, n (%)	825 (7.5)	526 (7.0)	271 (8.6)
RDCI≥3, n (%)	1020 (9.3)	640 (8.5)	350 (11.1)

*Missing information on serological status: 294 (2.7%).

DMARD, disease-modifying antirheumatic drug; RA, rheumatoid arthritis; RDCI, Rheumatic Disease Comorbidity Index.

Disease activity at RA diagnosis, overall and by the individual DAS28 components, is shown for all patients and divided in the different comorbidity categories in online supplemental table S9. Patients without any of the registered comorbidities tended to have the lowest disease activity measures, and also the lowest VAS pain (55 mm, IQR 34–73), whereas the patients with psychiatric diagnoses reported the highest VAS pain (64 mm, IQR 45–80) and PGA (62 mm, IQR 44–79).

### Failure to reach DAS28 remission by comorbidity burden

Higher comorbidity burden is associated with a higher risk of failure to reach DAS28 remission, both when measured by RDCI and when measured by the number of comorbidity categories (table 3), with up to 37% increased risk of failure to reach remission in individuals with ≥3 comorbidity categories, compared with patients with none of the comorbidities.

**Table 3:** Failure to reach DAS28 remission at 3-month and 6-month follow-up, by comorbidity burden

Measures of comorbidity burden	Proportion at 3 months n (%)	Proportion at 6 months n (%)	Not reaching DAS28 remission at 3 months n (%)	Not reaching DAS28 Remission at 6 months n (%)	Not reaching DAS28 remission at 3 months n=7643 RR (95% CI)*	Not reaching DAS28 remission at 6 months n=5391 RR (95% CI)*
RDCI: 0	5309 (69.5)	3785 (70.2)	2690 (50.7)	2160 (57.1)	Reference	Reference
RDCI: 1	1101 (14.4)	751 (13.9)	624 (56.7)	449 (59.8)	**1.15 (1.07 to 1.24)**	1.09 (0.99 to 1.20)
RDCI: 2	558 (7.3)	397 (7.4)	331 (59.3)	227 (57.2)	**1.27 (1.14 to 1.41)**	1.07 (0.95 to 1.21)
RDCI: ≥3	675 (8.8)	458 (8.5)	374 (55.4)	288 (62.9)	**1.19 (1.08 to 1.30)**	**1.31 (1.16 to 1.49)**
No of comorbidity categories: 0	3419 (44.7)	2401 (44.5)	1690 (49.4)	1332 (55.5)	Reference	Reference
No of comorbidity categories: 1	2166 (28.3)	1546 (28.7)	1146 (52.9)	895 (57.9)	**1.07 (1.02 to 1.14)**	1.07 (1.00 to 1.16)
No of comorbidity categories: 2	1248 (16.3)	891 (16.5)	710 (56.9)	543 (60.9)	**1.20 (1.11 to 1.29)**	**1.20 (1.09 to 1.31)**
No of comorbidity categories: ≥3	810 (10.6)	553 (10.3)	473 (58.4)	354 (64.0)	**1.27 (1.16 to 1.39)**	**1.37 (1.21 to 1.54)**

Statistically significant findings are in bold.

*Adjusted for sex and age.

DAS28, 28-joint Disease Activity Score; RDCI, Rheumatic Disease Comorbidity Index; RR, relative risk.

### Failure to reach DAS28 remission in relation to comorbidity categories

**Figure 2:**
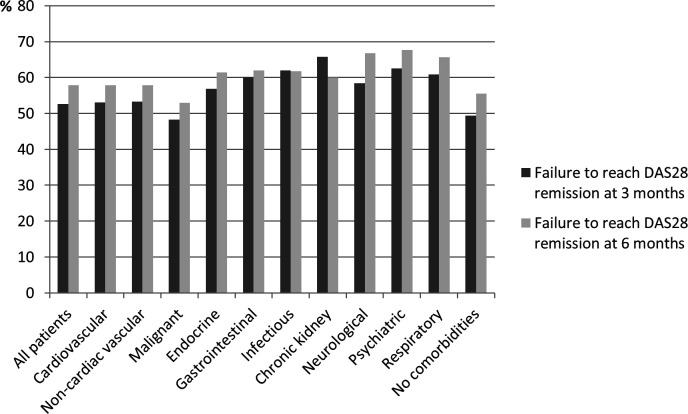
Proportion with failure to reach DAS28 remission at 3 and 6 months, by comorbidity categories. DAS28, 28-joint Disease Activity Score.

When assessing crude proportions, a total of 53% (n=4019/7643) of the patients had not reached DAS28 remission at 3 months, and 58% (n=3124/5391) at 6 months (figure 2 and online supplemental table S10). The proportion of patients who failed to reach DAS28 remission at 3 months was the highest (66%, n=25/38) among the patients with CKD and the lowest in the patients with a history of malignant disease (48%, n=154/319). Patients with a psychiatric comorbidity were most likely to fail to reach DAS28 remission at 6 months (68%, n=569/840). The proportion of patients with missing information on DAS28 remission at 3 months was 31% (n=3358), varying from 29% among patients with no reported comorbidities (n=1385) to 38% among patients with CKD (n=23). At 6 months, the degree of missing was 51% in total (n=5610), varying from 49% among patients with neurological (n=378) and psychiatric diseases (n=820) to 67% of those with CKD (n=41). There were 1882 patients (17%) who lacked information on DAS28 remission at both time points.

As seen in table 4, when adjusting for sex and age, and with all comorbidity categories incorporated in the modified Poisson regression, patients with endocrine (relative risk, RR 1.08, 95% CI 1.01 to 1.15), gastrointestinal (RR 1.16, 95% CI 1.03, 1.30), infectious (RR 1.21, 95% CI 1.06 to 1.38), psychiatric (RR 1.24, 95% CI 1.15 to 1.35) and respiratory comorbidities (RR 1.16, 95% CI 1.01 to 1.32) were at increased risk of not achieving DAS28 remission at 3 months, compared with patients without the comorbidity in question. Similar findings were observed in a full model with further adjustment for serological status, smoking, glucocorticoid treatment at onset, educational level and calendar period, as well as models with each comorbidity category analysed separately (table 4).

**Table 4:** Relative risk of failure to reach DAS28 remission at 3-month and 6-month follow-up in patients with early RA starting MTX in DMARD monotherapy, by comorbidity categories

	n (%) with data on DAS28 remission	Crude,* n of observations 3m=7643 6m=5391 RR (95% CI)	Adjusted for sex and age,* n of observations 3m=7643 6m=5391 RR (95% CI)	Full model,* n of observations 3m=5417 6m=3933 RR (95% CI)	Each comorbidity analysed separately,† adjusted for sex and age, n of observations 3m=7643 6m=5391 RR (95% CI)
Cardiovascular, 3m	636 (67.5)	0.96 (0.88 to 1.05)	1.02 (0.93 to 1.12)	0.99 (0.88 to 1.11)	1.09 (1.00 to 1.19)
Cardiovascular, 6m	394 (41.8)	0.98 (0.86 to 1.11)	1.08 (0.95 to 1.23)	1.05 (0.90 to 1.22)	**1.15 (1.02 to 1.30)**
Non-cardiac vascular, 3m	2128 (67.3)	0.97 (0.92 to 1.03)	1.01 (0.95 to 1.07)	1.01 (0.94 to 1.08)	**1.07 (1.01 to 1.13)**
Non-cardiac vascular, 6m	1422 (45.0)	0.96 (0.89 to 1.04)	1.02 (0.94 to 1.11)	1.04 (0.94 to 1.14)	**1.09 (1.01 to 1.17)**
Malignant, 3m	319 (68.3)	**0.89 (0.80 to 0.99)**	0.90 (0.81 to 1.00)	0.89 (0.78 to 1.02)	0.92 (0.83 to 1.03)
Malignant, 6m	208 (44.5)	0.87 (0.75 to 1.01)	0.90 (0.78 to 1.04)	1.04 (0.86 to 1.26)	0.92 (0.80 to 1.06)
Endocrine, 3m	1502 (67.2)	**1.10 (1.03 to 1.18)**	**1.08 (1.01 to 1.15)**	1.06 (0.98 to 1.15)	**1.11 (1.04 to 1.18)**
Endocrine, 6m	1079 (48.3)	**1.11 (1.02 to 1.21)**	**1.10 (1.01 to 1.20)**	1.10 (0.99 to 1.21)	**1.13 (1.04 to 1.23)**
Gastrointestinal, 3m	461 (67.7)	**1.15 (1.03 to 1.29)**	**1.16 (1.03 to 1.30)**	1.10 (0.97 to 1.26)	**1.21 (1.08 to 1.36)**
Gastrointestinal, 6m	334 (49.0)	1.08 (0.94 to 1.24)	1.09 (0.95 to 1.26)	1.03 (0.88 to 1.21)	1.14 (0.99 to 1.31)
Infectious, 3m	387 (63.2)	**1.20 (1.05 to 1.37)**	**1.21 (1.06 to 1.38)**	**1.28 (1.08 to 1.51)**	**1.29 (1.13 to 1.47)**
Infectious, 6m	267 (43.6)	1.06 (0.90 to 1.24)	1.10 (0.94 to 1.28)	1.09 (0.91 to 1.32)	**1.18 (1.01 to 1.37)**
Chronic kidney, 3m	38 (62.3)	1.33 (0.86 to 2.05)	1.38 (0.89 to 2.16)	1.26 (0.77 to 2.07)	1.50 (0.95 to 2.35)
Chronic kidney, 6m	20 (32.8)	1.04 (0.60 to 1.82)	1.06 (0.60 to 1.86)	1.26 (0.60 to 2.66)	1.13 (0.65 to 1.98)
Neurological, 3m	529 (68.7)	**1.12 (1.01 to 1.25)**	1.11 (1.00 to 1.23)	1.12 (0.99 to 1.26)	**1.14 (1.03 to 1.26)**
Neurological, 6m	392 (50.9)	**1.26 (1.09 to 1.46)**	**1.25 (1.08 to 1.44)**	**1.26 (1.06 to 1.48)**	**1.28 (1.11 to 1.48)**
Psychiatric, 3m	1111 (66.9)	**1.28 (1.18 to 1.39)**	**1.24 (1.15 to 1.35)**	**1.25 (1.14 to 1.38)**	**1.27 (1.17 to 1.38)**
Psychiatric, 6m	820 (49.4)	**1.33 (1.20 to 1.48)**	**1.27 (1.15 to 1.41)**	**1.29 (1.14 to 1.45)**	**1.31 (1.18 to 1.45)**
Respiratory, 3m	353 (67.2)	**1.17 (1.03 to 1.34)**	**1.16 (1.01 to 1.32)**	**1.22 (1.03 to 1.44)**	**1.22 (1.07 to 1.39)**
Respiratory, 6m	259 (49.3)	**1.20 (1.01 to 1.43)**	1.18 (1.00 to 1.40)	1.18 (0.97 to 1.44)	**1.24 (1.05 to 1.47)**

DAS28 remission was calculated on both DAS28 and DAS28-CRP. Full model: adjusted for sex, age, serological status, glucocorticoid use at RA diagnosis, educational level and calendar period. Statistically significant findings in bold.

*All comorbidities were incorporated in the same modified Poisson regression model.

†Each comorbidity category analysed in a separate model, that is, not adjusted for the other comorbidity categories.

CRP, C reactive protein; DAS28, 28-joint Disease Activity Score; DMARD, disease-modifying antirheumatic drug; m, month; MTX, methotrexate; RA, rheumatoid arthritis; RR, relative risk.

### Failure to reach other remission outcomes in relation to comorbidity categories

For the secondary remission outcomes; ACR/EULAR Boolean remission, SDAI/CDAI remission, EULAR good response (vs moderate/no) and no swollen joints; the RRs of not reaching remission were similar to the results regarding DAS28, both at the 3-month and 6-month follow-up visit (online supplemental tables S11–S15). Psychiatric and neurological comorbidities were the entities most consistently associated with failure to reach remission. This observation was true also for remission measures consisting of only objective criteria (no swollen joints), although with a less clear association.

### Failure to reach DAS28 remission in relation to comorbidity categories, stratified by sex and serological status

The RRs of failure to reach DAS28 remission were similar among females and males, although a larger number of the associations evaluated reached statistical significance among males (online supplemental table S16). The RR of DAS28 remission failure in patients with psychiatric diagnoses was somewhat higher in the seropositive group (RR 1.33, 95% CI 1.15 to 1.55) compared with the smaller seronegative group (RR 1.17, 95% CI 0.95 to 1.44, both at 6 months, online supplemental table S15).

## Discussion

In this nationwide register-based study on patients with early RA, treated with MTX in monotherapy as first DMARD, we found that a higher comorbidity burden and several comorbid conditions were associated with increased risk of failure to reach remission at 3 and 6 months after RA diagnosis. The results were coherent across the different remission measures and time points under study; the strongest associations were observed among patients with psychiatric comorbidities, who also reported the highest pain and PGA scores.

RA disease activity is assessed partly by composite indices, including subjective measures, which might be affected by psychiatric ill-being and thus be a possible explanation for the lower remission rates for these patients. This notion was supported by results from a Norwegian study, where questionnaire-based depression and anxiety at RA diagnosis were inversely associated with reaching RA remission at 3 and 6 months, the association being statistically significant only for the subjective measures; joint pain, PGA, EGA and TJC, not SJC or the inflammatory parameters.^29^ Results in this study were, however, also significant for the objective secondary outcome measure of no swollen joints. Functional capacity (HAQ) is influenced by comorbid conditions.^16 30^ Subjective disease activity measures, such as PGA, have previously been shown to be the most common limiting factor for reaching remission.^31^ Even though rheumatologists do not base their treatment decisions only on composite indices, subjective features can influence the general assessment of disease activity. The effect that comorbidities can have on disease activity and remission scores can also be a factor that has to be considered in, for example, randomised clinical trials evaluating the effect of new DMARDs. In several studies, higher age is associated with RA remission failure.^32–34^ Older patients have more comorbid conditions and general frailty can affect the subjective disease activity measures. Older patients can also be more prone to adverse events and be less intensively treated.^8 35 36^ We adjusted for age, and in the full model also for other relevant confounders, yet most findings remained, indicating that the results cannot solely be attributed to age.

Limitations of our study include that, to avoid the effects of comorbidities related to the choice of initial DMARD, we restricted our study to patients starting MTX as DMARD monotherapy. Our results are thus generalisable to this large group of patients but not necessarily to patients who start other DMARDs following RA diagnosis. Many patients receive MTX with concomitant glucocorticoids in early RA, also in our study, and the higher risk of remission failure at 6 months could partly be explained by discontinuation or lower dose of glucocorticoids at this time point. We could not in a reliable way assess the glucocorticoid dose at follow-up and, therefore, only adjusted for glucocorticoid use at the start of MTX in the full regression model. The results remained significant after this adjustment. The proportion of patients with missing information on DAS28 remission was rather large, with 31% missing data at 3 months and 51% at 6 months, thus raising the possibility of attrition bias. This is unfortunately a common problem for studies based on real-life/register data; patients do not come back at the planned time points for follow-up, or visits are not registered with all components of the composite disease measures in the quality register. It was not possible to assess if there were differences in remission among the patients with and without registered follow-up visits. However, the proportion with missing information was rather similar across the different comorbidity categories, with the exception of a higher degree of missing among patients with CKD, a comorbidity category consisting of few patients. We grouped our studied comorbidities in categories in order to reduce the risk with multiple testing, while still being able to study a large number of comorbidities. However, this grouping could cause problems if the included conditions affected remission in opposing directions. Lastly, comorbidity information was mainly retrieved from specialised care (and from PDR for all patients), which could to some extent exclude milder comorbid conditions, cared for in primary care. For example, the prevalence of CKD was lower than expected, possibly due to underreporting of mild CKD in the ICD-10 codes. The patients with CKD had the highest proportion of not reaching DAS28 remission, but in absolute number they were relatively few. We have previously shown that patients with CKD are treated with MTX to a lesser extent, which could have contributed to the low number of patients with CKD in this study.^8^

The main strengths of our study include being the first investigation of the association of a broad range of comorbidities, comorbidity burden and remission in patients with early RA treated with MTX in DMARD monotherapy. It is in addition a large study, with disease activity measures, comorbidity and prescription data from nationwide registers, which reduces the risk of selection and recall bias. Moreover, in order to focus on the effect of comorbidities, we only included patients with new-onset RA starting MTX in DMARD monotherapy, whereas most previous studies on comorbidities and RA remission have included patients with differences in both disease duration and antirheumatic treatment, factors that can also affect remission.^11 13 14 16–18^ The role of prompt and effective treatment on RA outcome has also been emphasised in RA, why we focused on follow-up already after 3 and 6 months. We were furthermore able to adjust for important confounders that could affect both presence of comorbidities and remission measures (ie, age, smoking, educational level, serological status).

RA is the most common inflammatory joint disease, with great burden for the individual patient as well as society if remission is not achieved. The impact of comorbidities in RA has gained increased attention in the past decades, however, most focus has been on established RA. In this study, we examined how comorbidities at RA onset associate with response to first-line treatment: MTX in DMARD monotherapy. Several comorbidities, in particular psychiatric diseases, and the overall comorbidity burden, were associated with failure to reach remission in early RA. This study shows that comorbidities may be important to consider when assessing treatment outcomes in early RA.

## Data Availability

Data are available on reasonable request. Requests for data should be directed to the senior author but will be conditioned on the legal premises under which they were collected.
