# Supplementary material for: Comorbidities and chance of remission in patients with early rheumatoid arthritis receiving methotrexate as first-line therapy: a Swedish observational nationwide study
Source: RMD Open. 2023 Dec 20;9(4):e003714. doi: 10.1136/rmdopen-2023-003714 (PMC10748957; doi:10.1136/rmdopen-2023-003714)
Supplement: Supplementary data [file rmdopen-2023-003714supp001.pdf]

Supplementary material

Supplemental table S1. List of registers used

Register	Description
**Education register (UREG)**	Founded in 1985 by Statistics Sweden (SCB). Covers highest level of educational attainment for each citizen.
**National Cancer Register**	Founded in 1958. Covers the whole population. Newly detected cancer (diagnosed at clinical, morphological or other laboratory examinations or autopsies) are compulsory to report.
**National Patient Register (NPR)**	Founded in the 1960s. Mandatory participation from all counties since 1984. Covers all in-patient care in Sweden since 1987. Since 2001 it also covers out-patient visits from specialized care from both private and public care-givers. Primary health care is not included.
**National Prescribed Drug Register (PDR)**	Founded in July 2005. Contains all prescribed drugs dispensed at pharmacies.
**Swedish Rheumatology Quality Register (SRQ)**	Founded in 1995. Covers information on diagnosis/ inclusion date, symptom duration, disease activity and treatment.
**Total Population Register (RTB)**	Founded in 1968 by Statistics Sweden (SCB). Covers information on the whole population concerning birth, death, area of residence, immigration, emigration etc.

Supplemental table S2. ICD-10 and ATC codes used for exclusion of patients*.

Diagnoses	ICD-10 codes
Rheumatoid arthritis	M05, M06.0, M06.2-M06.3, M06.8-M06.9, M12.3
Other arthritis	M13
Psoriatic arthritis	L40.5, M07.0, M07.1, M07.3
Ankylosing spondylitis	M45
Juvenile arthritis	M08-M09
Inflammatory spondylopathies	M46.0-M46.1, M46.8-M46.9
SLE	M32.0-M32.1, M32.8-M32.9
PMR	M35.3

Drugs	ATC codes
Abatacept	L04AA24
Adalimumab	L04AB04
Anakinra	L04AC03
Apremilast	L04AA32
Baricitinib	L04AA37
Belimumab	L04AA26
Canakinumab	L04AC08
Certolizumab pegol	L04AB05
Etanercept	L04AB01
Golimumab	L04AB06
Chloroquine	P01BA01
Leflunomide	L04AA13
Methotrexate	L04AX03
Sodium aurothiomalate	M01CB01
Hydroxychloroquine	P01BA02
Auranofin	M01CB03
Secukinumab	L04AC10
Sulfasalazine	A07EC01
Tocilizumab	L04AC07
Tofacitinib	L04AA29
Ustekinumab	L04AC05

*Patients were excluded if they had any of these diagnoses registered >12 months before the index date/methotrexate start or any of these drugs prescribed >1 month before the index date/methotrexate start in SRQ (Swedish Rheumatology Quality register). ICD-10, International Classification of Diseases, 10th revision; ATC, Anatomical Therapeutic Chemical Classification; SLE, systemic lupus erythematosus; PMR, polymyalgia rheumatica.

Supplemental table S3. Comorbidity categories and diagnoses with corresponding ICD-10 and/or ATC codes used to capture each comorbidity.

Comorbidity categories	Diagnoses	ICD-10 codes	ATC codes
**Cardiovascular**	Ischaemic heart disease	I20-I25	
	Acute coronary syndrome	I200, 121-122	
	Heart failure	I50	
	Atrial fibrillation	I48	
**Non-cardiac vascular**	Peripheral vascular diseases	I65-I66, I70-I72, I739, I74	
	Lipid lowering drugs		C10
	Stroke	I60-I64	
	Transient ischaemic attack	G45	
	Thromboembolic venous disease	I26, I80-I82	
	Hypertension	I10-I15	
**Respiratory**	Chronic obstructive pulmonary disease	J43-J44	
	Chronic interstitial pulmonary disease	J84	
	Asthma	J45	
**Gastrointestinal**	Inflammatory bowel disease	K50-K51	
	Esophageal, gastric and duodenal disease	K20-K23, K25-K27, K29	
	Biliary disease	K80-K81	
	Chronic liver disease	K70-K76	
**Psychiatric**	Depression	F32-F33	
	Antidepressant drugs		N06A
	Anxiety disorder	F40-F41, F43	
	Psychosis	F20-F29	
	Dementia (antidementia drugs)	F00-F03, G30-G31	N06D
**Chronic kidney**	Chronic kidney disease	I12-I13, N03-N05, N07, N18, Q61	
**Infectious**	Hospitalization due to infectious diseases	A00-B99, G00-G02, G042 G05-G07, H66-H67, J00-J22, J32, J340, J36, J838, J390-J391, J85-J86, L00-L08, M00-M01, M462-M465, M86, N10, N300, N390	
**Endocrine**	Type1 diabetes (insulin)*	E10	A10A
	Type 2 diabetes (glucose-lowering drugs)ɫ	E11	A10B
	Thyroid disease (thyroid and antithyroid preparations)	E038-E039, E050-E053, E055-E059	H03A, H03B
**Malignant**	Cancer	C00-C97, D00-D09	
**Neurological**	Parkinson's disease	G20	
	Multiple sclerosis	G35	
	Epilepsy	G40-G41	
	Polyneuropathies and nerve, nerve root and plexus disorders	G50-G59, G60-G65	
**Fractures‡**	Fractures of hip/spine/leg	M485, M489, M800A, M800E-F, M800J-K, M843E-G, S120-S127, S220-S221,	

S32, T021, T08, S72, S821-S824, S827

*For considered as having type 1 diabetes you had to have the E10 diagnosis, no E11 diagnosis, prescription of insulin and no prescription of glucose-lowering drugs.
łFor considered as having type 2 diabetes you had to have the E11 diagnosis or prescription of glucose-lowering drugs and no E10 diagnosis.
‡Information on fractures was only used for the Rheumatic Disease Comorbidity Index.
ICD-10, International Classification of Diseases 10th revision; ATC, Anatomical Therapeutic Chemical Classification.

Supplemental table S4. Rheumatic Disease Comorbidity Index.

**Comorbidity groups**	Lung disease<br>Heart Attack<br>Other Cardiovascular disease<br>Stroke<br>Hypertension<br>Diabetes<br>Fracture (of hip/spine/leg)<br>Depression<br>Cancer<br>Gastrointestinal ulcer<br>Other stomach problems
**Formula**	2 x lung disease + [2 x (heart attack, other cardiovascular disease, or stroke), or 1 x hypertension] + fracture + depression + diabetes + cancer + (ulcer or stomach problem)

See methods and reference 21-23.

Supplemental table S5. Definitions of outcome measures

OUTCOME	DEFINITION
	**Remission**
**DAS28 REMISSION**	DAS28 <2.6. DAS28= 0.56 x √(TJC28)+0.28 x √(SJC28)+ 0.014 x PGA + 0.70 x ln(ESR)
**DAS28-CRP REMISSION**	DAS28-CRP <2.4. DAS28-CRP= 0.56 x √(TJC28) + 0.28 x √(SJC28)+ 0.014 x PGA + 0.36 x ln(CRP+1) + 0.96
**ACR/EULAR BOOLEAN REMISSION**	TJC28 ≤1, SJC28≤1, PGA (0-10) ≤1 and CRP ≤1
**SDAI REMISSION**	SDAI ≤3.3. SDAI= SJC28 + TJC28 + PGA (0-10)+ EGA (0-10) + CRP
**CDAI REMISSION**	CDAI ≤2.8. CDAI= SJC28 + TJC28 + PGA (0-10)+ EGA (0-10)
**NO SWOLLEN JOINTS**	SJC28 = 0
	**Response**
**EULAR RESPONSE**	Either fulfilling a. or b.
	a. DAS28_0 - DAS28_x ≥1.2 and DAS28_x ≤3.2
	b. DAS28-CRP_0 - DAS28-CRP_x ≥1.2 and DAS28_x ≤3.2
	where _0 denotes the start of treatment and _x denotes the month for evaluation

Table adapted from Westerlind et al., Remission, response, retention and persistence to treatment with disease-modifying agents in patients with rheumatoid arthritis: a study of harmonised Swedish, Danish and Norwegian cohorts. RMD Open. 2023 Sep;9(3):e003027, supplemental table S2.

DAS28, disease activity score (28 joints); CRP, C-reactive protein (mg/dL); SDAI, simplified disease activity index; CDAI, clinical disease activity index; ACR/EULAR, American College of Rheumatology/European Alliance of Associations for Rheumatology; TJC, tender joint count (28 joints); SJC, swollen joint count (28 joints); PGA, patient global assessment; ESR, erythrocyte sedimentation rate (mm); EGA, evaluator global assessment.

Supplemental table S6. Comorbidity categories and comorbidity burden in RA patients initiating methotrexate as the only DMARD at RA diagnosis, overall and by sex

	All RA at diagnosis n=11 001	Females n=7419	Males n=3582
Cardiovascular, n (%)	942 (8.6)	411 (5.5)	531 (14.8)
Non-cardiac vascular, n (%)	3163 (28.8)	1823 (24.6)	1340 (37.4)
Malignant, n (%)	467 (4.3)	293 (4.0)	174 (4.9)
Endocrine, n (%)	2234 (20.3)	1605 (21.6)	629 (17.6)
Gastrointestinal, n (%)	681 (6.2)	452 (6.1)	229 (6.4)
Infectious, n (%)	612 (5.6)	385 (5.2)	227 (6.3)
Chronic kidney disease, n (%)	61 (0.6)	27 (0.4)	34 (1.0)
Neurological, n (%)	770 (7.0)	537 (7.2)	233 (6.5)
Psychiatric, n (%)	1660 (15.1)	1318 (17.8)	342 (9.6)
Respiratory, n (%)	525 (4.8)	358 (4.8)	167 (4.7)
No comorbidity, n (%)	4804 (43.7)	3265 (44.0)	1539 (43.0)
RDCI: 0, n (%)	7554 (68.7)	5324 (71.8)	2230 (62.3)
RDCI: 1, n (%)	1602 (14.6)	1093 (14.7)	509 (14.2)
RDCI: 2, n (%)	825 (7.5)	479 (6.5)	346 (9.7)
RDCI≥3, n (%)	1020 (9.3)	523 (7.1)	497 (13.9)

RA, rheumatoid arthritis; DMARD, disease-modifying antirheumatic drugs; RDCI, Rheumatic Disease Comorbidity Index.

Supplemental table S7. Comorbidity categories and comorbidity burden in RA patients initiating methotrexate as the only DMARD at RA diagnosis, overall and by age groups

	**All RA at diagnosis n=11 001**	**18-49 years n=2540**	**50-74 years n=6691**	**≥75 years n=1770**
Cardiovascular, n (%)	942 (8.6)	6 (0.2)	548 (8.2)	388 (21.9)
Non-cardiac vascular, n (%)	3163 (28.8)	117 (4.6)	2105 (31.5)	941 (53.2)
Malignant, n (%)	467 (4.3)	49 (1.9)	294 (4.4)	124 (7.0)
Endocrine, n (%)	2234 (20.3)	340 (13.4)	1403 (21.0)	491 (27.7)
Gastrointestinal, n (%)	681 (6.2)	95 (3.7)	427 (6.4)	159 (9.0)
Infectious, n (%)	612 (5.6)	58 (2.3)	360 (5.4)	194 (11.0)
Chronic kidney disease, n (%)	61 (0.6)	5 (0.2)	34 (0.5)	22 (1.2)
Neurological, n (%)	770 (7.0)	137 (5.4)	517 (7.7)	116 (6.6)
Psychiatric, n (%)	1660 (15.1)	405 (15.9)	1000 (15.0)	255 (14.4)
Respiratory, n (%)	525 (4.8)	62 (2.4)	328 (4.9)	135 (7.6)
No comorbidity, n (%)	4804 (43.7)	1616 (63.6)	2746 (41.0)	442 (25.0)
RDCI: 0, n (%)	7554 (68.7)	2166 (85.3)	4526 (67.6)	862 (48.7)
RDCI: 1, n (%)	1602 (14.6)	266 (10.5)	1021 (15.3)	315 (17.8)
RDCI: 2, n (%)	825 (7.5)	87 (3.4)	546 (8.2)	192 (10.9)
RDCI≥3, n (%)	1020 (9.3)	21 (0.8)	598 (8.9)	401 (22.7)

RA, rheumatoid arthritis; DMARD, disease-modifying antirheumatic drugs; RDCI, Rheumatic Disease Comorbidity Index.

Supplemental table S8. Number of early RA patients initiating methotrexate as the only DMARD, with a visit registered in SRQ at RA diagnosis, at 3 and 6 months follow-up

	RA diagnosis	3 months	6 months
All patients, n (%)	10 088 (91.7)	8273 (75.2)	5924 (53.8)
Cardiovascular, n (%)	849 (90.1)	700 (74.3)	447 (47.5)
Non-cardiac vascular, n (%)	2891 (91.4)	2313 (73.1)	1609 (50.9)
Malignant, n (%)	437 (93.6)	340 (72.8)	230 (49.3)
Endocrine, n (%)	2002 (89.6)	1635 (73.2)	1196 (53.5)
Gastrointestinal, n (%)	630 (92.5)	500 (73.4)	361 (53.0)
Infectious, n (%)	560 (91.5)	425 (69.4)	309 (50.5)
Chronic kidney, n (%)	57 (93.4)	42 (68.9)	26 (42.6)
Neurological, n (%)	708 (91.9)	578 (75.1)	431 (56.0)
Psychiatric, n (%)	1514 (91.2)	1222 (73.6)	910 (54.8)
Respiratory, n (%)	479 (91.2)	395 (75.2)	293 (55.8)
No comorbidity, n (%)	4430 (92.2)	3680 (76.6)	2620 (54.5)

RA, rheumatoid arthritis; DMARD, disease-modifying antirheumatic drugs; SRQ, Swedish Rheumatology Quality register.

Supplemental table S9. Disease activity at RA diagnosis, by serological status and comorbidity categories

	DAS28 median (IQR)	DAS28-CRP median (IQR)	SJC median (IQR)	TJC median (IQR)	CRP median (IQR)	ESR median (IQR)	PGA VAS median (IQR)	HAQ median (IQR)	Pain VAS median (IQR)
All	5.1 (4.2-6.0)	4.7 (3.9-5.6)	7 (4-11)	6 (3-10)	12 (5-30)	28 (15-46)	54 (34-73)	1.0 (0.6-1.5)	57 (35-75)
Seropositive	5.1 (4.2-5.9)	4.6 (3.8-5.4)	6 (3-10)	6 (3-10)	11 (5-27)	28 (15-47)	53 (33-73)	1.0 (0.5-1.4)	58 (35-75)
Seronegative	5.3 (4.4-6.2)	5.0 (4.2-5.8)	8 (5-13)	8 (4-12)	14 (5-35)	27 (14-45)	53 (35-72)	1.0 (0.6-1.5)	56 (35-74)
Cardiovascular	5.4 (4.6-6.1)	5.0 (4.2-5.7)	7 (4-12)	7 (4-11)	15 (6-36)	33 (19-53)	54 (35-73)	1.1 (0.6-1.6)	58 (35-74)
Non-cardiac vascular	5.3 (4.4-6.1)	4.9 (4.0-5.6)	7 (4-11)	6 (3-11)	14 (5-34)	32 (18-52)	54 (36-74)	1.0 (0.6-1.5)	59 (36-75)
Cancer	5.3 (4.4-6.1)	4.8 (4.0-5.6)	7 (4-11)	6 (3-10)	14 (5-33)	30 (19-49)	51 (34-73)	1.1 (0.6-1.5)	56 (34-75)
Endocrine	5.3 (4.4-6.1)	4.9 (4.0-5.7))	7 (4-11)	7 (3-11)	12 (5-30)	30 (18-49)	55 (39-75)	1.1 (0.6-1.6)	60 (40-78)
Gastrointestinal	5.2 (4.3-6.0)	4.8 (4.1-5.6)	7 (3-11)	7 (3-11)	13 (5-28)	28 (14-44)	57 (41-75)	1.1 (0.8-1.6)	63 (45-76)
Infectious	5.4 (4.5-6.2)	5.0 (4.1-5.7)	7 (4-11)	7 (4-11)	15 (6-40)	33 (19-53)	57 (38-75)	1.1 (0.8-1.8)	58 (36-76)
Chronic kidney disease	5.5 (4.6-6.1)	4.8 (4.2-5.4)	8 (5-11)	7 (3-10)	11 (5-25)	36 (21-67)	51 (33-65)	1.1 (0.6-1.6)	60 (31-80)
Neurological	5.2 (4.2-6.1)	4.8 (4.0-5.7)	7 (4-11)	7 (3-12)	10 (5-24)	25 (13-40)	58 (38-76)	1.0 (0.6-1.6)	61 (40-78)
Psychiatric	5.3 (4.4-6.2)	4.8 (4.1-5.7)	6 (4-10)	7 (4-11)	11 (4-25)	27 (14-43)	62 (44-79)	1.1 (0.8-1.8)	64 (45-80)
Respiratory	5.3 (4.3-6.1)	4.9 (3.9-5.6)	7 (3-10)	7 (3-11)	14 (5-32)	32 (18-49)	54 (37-75)	1.1 (0.6-1.8)	59 (36-75)
No comorbidity	5.0 (4.1-5.9)	4.7 (3.8-5.5)	6 (3-10)	6 (3-10)	11 (5-29)	25 (13-44)	51 (31-70)	0.9 (0.5-1.4)	55 (34-73)

RA, rheumatoid arthritis; DAS28, 28-joint Disease Activity Score; SJC, swollen joint count (28 joints); TJC, tender joint count (28 joints); CRP, C-reactive protein (mg/L); ESR, erythrocyte sedimentation rate (mm); PGA, patient's global assessment; VAS, visual analog scale; HAQ, health assessment questionnaire; IQR, interquartile range.

Supplemental table S10. Proportion with failure to reach DAS28 remission at 3 and 6 months after initiating methotrexate monotherapy in early RA, by comorbidity categories

	Not reaching DAS28 remission at 3 months	Missing at 3 months	Not reaching DAS28 remission at 6 months	Missing at 6 months
All patients, n (%)	4019/7643 (52.6)	3358/11 001 (30.5)	3124/5391 (57.9)	5610/11001 (51.0)
Cardiovascular, n (%)	338/636 (53.1)	306/942 (32.5)	228/394 (57.9)	548/942 (58.2)
Non-cardiac vascular, n (%)	1135/2128 (53.3)	1035/3163 (32.7)	822/1422 (57.8)	1741/3163 (55.0)
Malignant, n (%)	154/319 (48.3)	148/467 (31.7)	110/208 (52.9)	259/467 (55.5)
Endocrine, n (%)	853/1502 (56.8)	732/2234 (32.8)	663/1079 (61.4)	1155/2234 (51.7)
Gastrointestinal, n (%)	277/461 (60.1)	220/681 (32.3)	207/334 (62.0)	347/681 (51.0)
Infectious, n (%)	240/387 (62.0)	225/612 (36.8)	165/267 (61.8)	345/612 (56.4)
Chronic kidney disease, n (%)	25/38 (65.8)	23/61 (37.7)	12/20 (60.0)	41/61 (67.2)
Neurological, n (%)	309/529 (58.4)	241/770 (31.3)	262/392 (66.8)	378/770 (49.1)
Psychiatric, n (%)	694/1111 (62.5)	549/1660 (33.1)	569/840 (67.7)	820/1660 (49.4)
Respiratory, n (%)	215/353 (60.9)	172/525 (32.8)	170/259 (65.6)	266/525 (50.7)
No comorbidities, n (%)	1690/3419 (49.4)	1385/4804 (28.8)	1332/2401 (55.5)	2403/4804 (50.0)

DAS28, 28-joint Disease Activity Score; RA, rheumatoid arthritis.

Supplemental table S11. Relative risk of failure to reach Boolean remission at 3 and 6 months after RA diagnosis, by comorbidity categories

	n (%) with data on Boolean remission	Crude, n of observations 3m=7595 6m=5356 RR (95% CI)	Adjusted for sex and age, n of observations 3m=7595 6m=5356 RR (95% CI)	Full model, n of observations 3m=5387 6m=3905 RR (95% CI)
Cardiovascular, 3m	630 (66.9)	1.17 (0.97, 1.41)	**1.26 (1.04, 1.52)**	**1.28 (1.02, 1.61)**
Cardiovascular, 6m	392 (41.6)	0.97 (0.77, 1.23)	1.10 (0.87, 1.40)	0.91 (0.69, 1.18)
Non-cardiac vascular, 3m	2113 (66.8)	0.92 (0.83, 1.03)	0.99 (0.89, 1.11)	0.96 (0.84, 1.10)
Non-cardiac vascular, 6m	1415 (44.7)	0.90 (0.78, 1.04)	0.98 (0.84, 1.13)	1.07 (0.89, 1.28)
Malignant, 3m	317 (67.9)	0.85 (0.69, 1.04)	0.87 (0.71, 1.06)	0.83 (0.66, 1.06)
Malignant, 6m	208 (44.5)	1.05 (0.77, 1.43)	1.09 (0.80, 1.48)	1.14 (0.79, 1.65)
Endocrine, 3m	1490 (66.7)	**1.16 (1.02, 1.31)**	**1.14 (1.01, 1.29)**	1.16 (1.00, 1.35)
Endocrine, 6m	1073 (48.0)	**1.25 (1.07, 1.47)**	**1.24 (1.05, 1.45)**	**1.24 (1.03, 1.51)**
Gastrointestinal, 3m	458 (67.3)	**1.27 (1.02, 1.59)**	**1.29 (1.04, 1.61)**	1.23 (0.96, 1.58)
Gastrointestinal, 6m	331 (48.6)	1.07 (0.83, 1.38)	1.09 (0.85, 1.40)	1.03 (0.77, 1.37)
Infectious, 3m	383 (62.6)	**1.40 (1.08, 1.82)**	**1.42 (1.10, 1.85)**	1.30 (0.96, 1.75)
Infectious, 6m	265 (43.3)	1.08 (0.81, 1.44)	1.13 (0.85, 1.50)	1.08 (0.78, 1.50)
Chronic kidney, 3m	38 (62.3)	1.01 (0.53, 1.94)	1.05 (0.54, 2.03)	0.90 (0.46, 1.75)
Chronic kidney, 6m	20 (32.8)	0.88 (0.36, 2.16)	0.90 (0.35, 2.27)	0.95 (0.31, 2.86)
Neurological, 3m	528 (68.6)	**1.25 (1.03, 1.53)**	**1.25 (1.02, 1.53)**	1.26 (1.00, 1.59)
Neurological, 6m	390 (50.6)	**1.38 (1.06, 1.80)**	**1.37 (1.05, 1.78)**	**1.39 (1.02, 1.88)**
Psychiatric, 3m	1103 (66.4)	**1.59 (1.36, 1.86)**	**1.53 (1.30, 1.79)**	**1.63 (1.35, 1.98)**
Psychiatric, 6m	840 (50.6)	**1.58 (1.30, 1.93)**	**1.50 (1.23, 1.82)**	**1.62 (1.27, 2.05)**
Respiratory, 3m	348 (66.3)	1.16 (0.91, 1.48)	1.14 (0.90, 1.45)	1.27 (0.94, 1.73)
Respiratory, 6m	258 (49.1)	**1.46 (1.05, 2.05)**	**1.43 (1.02, 2.00)**	1.40 (0.96, 2.04)

All comorbidities were incorporated in the same modified Poisson regression model. Full model: adjusted for sex, age, serological status, smoking, glucocorticoid use at RA diagnosis, educational level, and calendar period. Significant findings in bold. RA, rheumatoid arthritis; RR, relative risk.

Supplemental table S12. Relative risk of failure to reach CDAI remission at 3 and 6 months after RA diagnosis, by comorbidity categories

	n (%) with data on CDAI	Crude, n of observations 3m=7190 6m=5282 RR (95% CI)	Adjusted for sex and age, n of observations 3m=7190 6m=5282 RR (95% CI)	Full model, n of observations 3m=5142 6m=3861 RR (95% CI)
Cardiovascular, 3m	594 (63.1)	1.04 (0.86, 1.26)	1.13 (0.93, 1.37)	1.13 (0.89, 1.43)
Cardiovascular, 6m	395 (41.9)	1.10 (0.86, 1.40)	1.25 (0.98, 1.59)	1.13 (0.85, 1.50)
Non-cardiac vascular, 3m	1997 (63.1)	1.05 (0.93, 1.18)	1.12 (0.99, 1.26)	1.09 (0.95, 1.26)
Non-cardiac vascular, 6m	1392 (44.0)	0.92 (0.79, 1.06)	0.99 (0.85, 1.15)	1.08 (0.90, 1.29)
Malignant, 3m	300 (64.2)	0.83 (0.67, 1.02)	0.85 (0.68, 1.05)	**0.77 (0.61, 0.98)**
Malignant, 6m	207 (44.3)	0.80 (0.62, 1.04)	0.83 (0.64, 1.08)	0.91 (0.66, 1.26)
Endocrine, 3m	1399 (62.6)	1.03 (0.91, 1.16)	1.02 (0.90, 1.15)	0.99 (0.85, 1.15)
Endocrine, 6m	1055 (47.2)	1.08 (0.93, 1.26)	1.07 (0.92, 1.24)	0.98 (0.82, 1.17)
Gastrointestinal, 3m	432 (63.4)	1.12 (0.90, 1.39)	1.14 (0.92, 1.42)	1.09 (0.85, 1.41)
Gastrointestinal, 6m	329 (48.3)	1.15 (0.89, 1.49)	1.17 (0.90, 1.51)	1.11 (0.83, 1.50)
Infectious, 3m	361 (59.0)	1.08 (0.85, 1.37)	1.10 (0.87, 1.39)	1.27 (0.94, 1.73)
Infectious, 6m	263 (43.0)	0.82 (0.64, 1.06)	0.87 (0.68, 1.11)	0.83 (0.62, 1.11)
Chronic kidney disease, 3m	35 (57.4)	0.90 (0.47, 1.74)	0.93 (0.48, 1.80)	0.74 (0.38, 1.47)
Chronic kidney disease, 6m	18 (29.5)	0.81 (0.33, 1.98)	0.80 (0.32, 1.98)	1.05 (0.28, 3.95)
Neurological, 3m	501 (65.1)	1.21 (0.98, 1.48)	1.21 (0.98, 1.48)	1.17 (0.92, 1.47)
Neurological, 6m	383 (49.7)	**1.70 (1.27, 2.29)**	**1.68 (1.25, 2.26)**	**1.64 (1.17, 2.29)**
Psychiatric, 3m	1030 (62.0)	**1.57 (1.33, 1.85)**	**1.52 (1.28, 1.79)**	**1.70 (1.38, 2.09)**
Psychiatric, 6m	819 (49.3)	**1.61 (1.32, 1.96)**	**1.52 (1.25, 1.85)**	**1.58 (1.25, 2.00)**
Respiratory, 3m	333 (63.4)	1.19 (0.92, 1.55)	1.18 (0.91, 1.53)	1.16 (0.85, 1.58)
Respiratory, 6m	251 (47.8)	**1.40 (1.01, 1.93)**	1.35 (0.98, 1.87)	1.40 (0.96, 2.04)

All comorbidities were incorporated in the same modified Poisson regression model. Full model: adjusted for sex, age, serological status, smoking, glucocorticoid use at RA diagnosis, educational level, and calendar period. Significant findings in bold. CDAI, clinical disease activity index; RA, rheumatoid arthritis; RR, relative risk.

Supplemental table S13. Relative risk of failure to reach SDAI remission at 3 and 6 months after RA diagnosis, by comorbidity categories

	**n (%) with data on SDAI**	**Crude, n of observations 3m=7222 6m=5208 RR (95% CI)**	**Adjusted for sex and age, n of observations 3m=7222 6m=5208 RR (95% CI)**	**Full model, n of observations 3m=5152 6m=3803 RR (95% CI)**
Cardiovascular, 3m	596 (63.2)	1.07 (0.90, 1.28)	1.15 (0.96, 1.37)	1.20 (0.96, 1.50)
Cardiovascular, 6m	384 (40.8)	1.09 (0.87, 1.37)	1.24 (0.99, 1.56)	1.13 (0.86, 1.47)
Non-cardiac vascular, 3m	2002 (63.3)	1.00 (0.90, 1.11)	1.06 (0.95, 1.18)	1.05 (0.92, 1.19)
Non-cardiac vascular, 6m	1371 (43.3)	0.93 (0.82, 1.07)	1.01 (0.88, 1.15)	1.11 (0.94, 1.32)
Malignant, 3m	298 (63.8)	0.90 (0.73, 1.10)	0.91 (0.74, 1.12)	0.82 (0.66, 1.04)
Malignant, 6m	204 (43.7)	0.87 (0.68, 1.12)	0.90 (0.70, 1.16)	0.95 (0.70, 1.29)
Endocrine, 3m	1419 (63.5)	1.04 (0.92, 1.16)	1.02 (0.91, 1.15)	0.99 (0.86, 1.14)
Endocrine, 6m	1041 (46.6)	1.09 (0.94, 1.25)	1.07 (0.93, 1.23)	0.99 (0.84, 1.17)
Gastrointestinal, 3m	434 (63.7)	1.20 (0.97, 1.48)	1.21 (0.99, 1.49)	1.14 (0.90, 1.45)
Gastrointestinal, 6m	324 (47.6)	1.21 (0.94, 1.54)	1.22 (0.96, 1.56)	1.21 (0.91, 1.62)
Infectious, 3m	362 (59.2)	1.12 (0.89, 1.40)	1.14 (0.91, 1.42)	1.21 (0.92, 1.61)
Infectious, 6m	260 (42.5)	0.82 (0.65, 1.03)	0.86 (0.69, 1.09)	0.85 (0.65, 1.11)
Chronic kidney, 3m	36 (59.0)	0.81 (0.47, 1.41)	0.84 (0.48, 1.47)	0.72 (0.39, 1.34)
Chronic kidney, 6m	18 (29.5)	0.92 (0.37, 2.25)	0.91 (0.37, 2.24)	1.19 (0.31, 4.54)
Neurological, 3m	503 (65.3)	**1.22 (1.01, 1.48)**	1.22 (1.00, 1.47)	1.22 (0.97, 1.52)
Neurological, 6m	380 (49.4)	**1.77 (1.34, 2.35)**	**1.75 (1.33, 2.32)**	**1.66 (1.21, 2.27)**
Psychiatric, 3m	1039 (62.6)	**1.51 (1.30, 1.76)**	**1.47 (1.26, 1.71)**	**1.59 (1.32, 1.92)**
Psychiatric, 6m	813 (49.0)	**1.58 (1.31, 1.89)**	**1.49 (1.24, 1.78)**	**1.59 (1.27, 1.98)**
Respiratory, 3m	333 (63.4)	1.11 (0.88, 1.39)	1.09 (0.87, 1.37)	1.18 (0.88, 1.58)
Respiratory, 6m	249 (47.4)	**1.37 (1.01, 1.84)**	1.32 (0.98, 1.78)	1.38 (0.97, 1.98)

All comorbidities were incorporated in the same modified Poisson regression model. Full model: adjusted for sex, age, serological status, smoking, glucocorticoid use at RA diagnosis, educational level, and calendar period. Significant findings in bold. SDAI, simplified disease activity index; RA, rheumatoid arthritis; RR, relative risk.

Supplemental table S14. Relative risk of failure to reach EULAR response at 3 and 6 months after RA diagnosis, by comorbidity categories

	n (%) with data on EULAR response	Crude, n of observations 3m=6555 6m=4816 RR (95% CI)	Adjusted for sex and age, n of observations 3m=6555 6m=4816 RR (95% CI)	Full model, n of observations 3m=5025 6m=3710 RR (95% CI)
Cardiovascular, 3m	529 (56.2)	0.98 (0.90, 1.06)	1.04 (0.96, 1.13)	1.01 (0.92, 1.12)
Cardiovascular, 6m	350 (37.2)	0.99 (0.88, 1.12)	1.08 (0.96, 1.21)	1.06 (0.93, 1.21)
Non-cardiac vascular, 3m	1804 (57.0)	**0.93 (0.88, 0.98)**	0.98 (0.93, 1.04)	0.97 (0.91, 1.04)
Non-cardiac vascular, 6m	1262 (39.9)	**0.88 (0.82, 0.94)**	0.95 (0.89, 1.02)	0.96 (0.88, 1.04)
Malignant, 3m	278 (59.5)	**0.88 (0.80, 0.97)**	0.91 (0.82, 1.00)	0.97 (0.86, 1.09)
Malignant, 6m	186 (39.8)	**0.86 (0.75, 0.99)**	0.90 (0.79, 1.03)	0.95 (0.81, 1.12)
Endocrine, 3m	1264 (56.6)	1.06 (1.00, 1.12)	1.05 (0.99, 1.12)	1.03 (0.97, 1.11)
Endocrine, 6m	939 (42.0)	**1.10 (1.01, 1.19)**	**1.11 (1.02, 1.20)**	1.07 (0.98, 1.17)
Gastrointestinal, 3m	399 (58.6)	**1.13 (1.02, 1.26)**	**1.14 (1.03, 1.27)**	1.10 (0.98, 1.23)
Gastrointestinal, 6m	310 (45.5)	1.12 (0.98, 1.28)	1.14 (1.00, 1.30)	1.06 (0.92, 1.23)
Infectious, 3m	322 (52.6)	**1.22 (1.08, 1.39)**	**1.24 (1.09, 1.40)**	**1.22 (1.05, 1.42)**
Infectious, 6m	235 (38.4)	1.09 (0.93, 1.27)	1.13 (0.97, 1.32)	1.11 (0.94, 1.32)
Chronic kidney, 3m	33 (54.1)	0.95 (0.71, 1.27)	0.95 (0.71, 1.27)	0.90 (0.66, 1.23)
Chronic kidney, 6m	19 (31.1)	1.56 (0.80, 3.04)	1.61 (0.82, 3.14)	1.67 (0.73, 3.82)
Neurological, 3m	443 (57.5)	1.06 (0.97, 1.16)	1.06 (0.96, 1.16)	1.03 (0.93, 1.14)
Neurological, 6m	352 (45.7)	**1.18 (1.04, 1.35)**	**1.18 (1.04, 1.35)**	**1.17 (1.01, 1.35)**
Psychiatric, 3m	958 (57.7)	**1.21 (1.13, 1.30)**	**1.19 (1.10, 1.27)**	**1.16 (1.07, 1.26)**
Psychiatric, 6m	746 (44.9)	**1.33 (1.21, 1.47)**	**1.29 (1.17, 1.43)**	**1.30 (1.16, 1.46)**
Respiratory, 3m	298 (56.8)	**1.14 (1.01, 1.28)**	1.13 (1.00, 1.28)	**1.25 (1.07, 1.45)**
Respiratory, 6 m	230 (43.8)	**1.24 (1.05, 1.47)**	**1.23 (1.04, 1.44)**	1.18 (0.98, 1.42)

All comorbidities were incorporated in the same modified Poisson regression model. Full model: adjusted for sex, age, serological status, smoking, glucocorticoid use at RA diagnosis, educational level, and calendar period. Significant findings in bold. EULAR response, EULAR good response compared with moderate/no response; RA, rheumatoid arthritis; RR, relative risk.

Supplemental table S15. Relative risk of failure to reach No swollen joints at 3 and 6 months after RA diagnosis, by comorbidity categories

	**n (%) with data on swollen joints**	**Crude, n of observations 3m=8028 6m=5608 RR (95% CI)**	**Adjusted for sex and age, n of observations 3m=8028 6m=5608 RR (95% CI)**	**Full model, n of observations 3m=5599 6m=4059 RR (95% CI)**
Cardiovascular, 3m	678 (72.0)	1.04 (0.94, 1.15)	1.07 (0.97, 1.18)	1.04 (0.92, 1.18)
Cardiovascular, 6m	416 (44.2)	0.98 (0.87, 1.10)	1.03 (0.92, 1.17)	1.00 (0.87, 1.15)
Non-cardiac vascular, 3m	2246 (71.0)	0.97 (0.91, 1.03)	0.99 (0.93, 1.05)	0.96 (0.89, 1.04)
Non-cardiac vascular, 6m	1497 (47.3)	0.94 (0.87, 1.01)	0.98 (0.91, 1.06)	0.98 (0.90, 1.07)
Malignant, 3m	335 (71.7)	0.90 (0.81, 1.01)	0.91 (0.81, 1.02)	**0.85 (0.74, 0.97)**
Malignant, 6m	219 (46.9)	0.89 (0.78, 1.03)	0.91 (0.79, 1.05)	1.02 (0.85, 1.22)
Endocrine, 3m	1571 (70.3)	1.04 (0.98, 1.11)	1.04 (0.97, 1.11)	1.05 (0.97, 1.14)
Endocrine, 6m	1135 (50.8)	1.01 (0.94, 1.09)	1.01 (0.93, 1.09)	0.99 (0.90, 1.09)
Gastrointestinal, 3m	488 (71.7)	0.99 (0.89, 1.09)	0.99 (0.89, 1.10)	0.93 (0.83, 1.05)
Gastrointestinal, 6m	344 (50.5)	1.06 (0.93, 1.21)	1.07 (0.94, 1.22)	1.01 (0.87, 1.18)
Infectious, 3m	416 (68.0)	1.11 (0.98, 1.25)	1.12 (0.99, 1.26)	1.09 (0.94, 1.27)
Infectious, 6m	283 (46.2)	0.95 (0.83, 1.09)	0.98 (0.85, 1.12)	1.00 (0.85, 1.17)
Chronic kidney, 3m	40 (65.6)	1.16 (0.77, 1.74)	1.16 (0.78, 1.75)	1.06 (0.67, 1.67)
Chronic kidney, 6m	23 (37.7)	1.04 (0.65, 1.66)	1.06 (0.66, 1.69)	1.04 (0.63, 1.71)
Neurological, 3m	556 (72.2)	**1.15 (1.03, 1.28)**	**1.15 (1.03, 1.28)**	1.14 (1.00, 1.29)
Neurological, 6m	405 (52.6)	**1.21 (1.06, 1.38)**	**1.20 (1.05, 1.38)**	**1.18 (1.01, 1.38)**
Psychiatric, 3m	1187 (71.5)	**1.11 (1.03, 1.19)**	**1.10 (1.02, 1.18)**	1.09 (0.99, 1.19)
Psychiatric, 6m	871 (52.5)	**1.14 (1.04, 1.25)**	**1.11 (1.01, 1.22)**	1.11 (1.00, 1.24)
Respiratory, 3m	383 (73.0)	1.08 (0.95, 1.22)	1.07 (0.95, 1.22)	**1.26 (1.06, 1.50)**
Respiratory, 6 m	270 (51.4)	0.98 (0.86, 1.13)	0.98 (0.85, 1.12)	0.97 (0.82, 1.14)

All comorbidities were incorporated in the same modified Poisson regression model. Full model: adjusted for sex, age, smoking, serological status, glucocorticoid use at RA diagnosis, educational level, and calendar period. Significant findings in bold. No swollen joint count was calculated on 28 joints. RA, rheumatoid arthritis; RR, relative risk.

Supplemental table S16. Relative risk of failure to reach DAS28 remission at 3 and 6 months after RA diagnosis, by comorbidity categories and sex

	n (%) of females with data on DAS28	Females, n of observations 3m=3714 6m=2706 RR (95% CI)	n (%) of males with data on DAS28	Males, n of observations 3m=1703 6m=1227 RR (95% CI)
Cardiovascular, 3m	274 (66.7)	1.04 (0.85, 1.27)	362 (68.2)	0.97 (0.84, 1.12)
Cardiovascular, 6m	153 (37.2)	1.28 (0.92, 1.77)	241 (45.4)	1.01 (0.85, 1.19)
Non-cardiac vascular, 3m	1226 (67.3)	1.03 (0.93, 1.14)	902 (67.3)	0.97 (0.87, 1.07)
Non-cardiac vascular, 6m	830 (45.5)	1.07 (0.94, 1.22)	592 (44.2)	0.97 (0.85, 1.11)
Malignant, 3m	208 (71.0)	0.91 (0.76, 1.09)	111 (63.8)	0.87 (0.72, 1.05)
Malignant, 6m	134 (45.7)	1.13 (0.85, 1.49)	74 (42.5)	0.96 (0.75, 1.24)
Endocrine, 3m	1077 (67.1)	1.03 (0.93, 1.13)	425 (67.6)	**1.16 (1.01, 1.33)**
Endocrine, 6m	781 (48.7)	1.06 (0.93, 1.20)	298 (47.4)	**1.19 (1.01, 1.40)**
Gastrointestinal, 3m	297 (65.7)	1.20 (1.00, 1.44)	164 (71.6)	0.97 (0.81, 1.17)
Gastrointestinal, 6m	222 (49.1)	0.98 (0.80, 1.19)	112 (48.9)	1.16 (0.91, 1.49)
Infectious, 3m	241 (62.6)	1.26 (1.00, 1.57)	146 (64.3)	**1.30 (1.01, 1.66)**
Infectious, 6m	159 (41.3)	1.22 (0.91, 1.62)	108 (47.6)	0.96 (0.76, 1.22)
Chronic kidney, 3m	13 (48.1)	0.91 (0.42, 1.94)	25 (73.5)	1.47 (0.77, 2.83)
Chronic kidney, 6m	11 (40.7)	0.91 (0.37, 2.23)	9 (26.5)	1.88 (0.59, 5.94)
Neurological, 3m	373 (69.5)	1.09 (0.94, 1.27)	156 (67.0)	1.18 (0.95, 1.47)
Neurological, 6m	284 (52.9)	1.17 (0.96, 1.41)	108 (46.4)	**1.50 (1.09, 2.05)**
Psychiatric, 3m	877 (66.5)	**1.24 (1.11, 1.38)**	234 (68.4)	**1.29 (1.07, 1.55)**
Psychiatric, 6m	684 (51.9)	**1.28 (1.11, 1.48)**	156 (45.6)	**1.29 (1.02, 1.64)**
Respiratory, 3m	246 (68.7)	1.21 (0.98, 1.50)	107 (64.1)	1.22 (0.93, 1.60)
Respiratory, 6m	183 (51.1)	1.15 (0.89, 1.49)	76 (45.5)	1.15 (0.86, 1.55)

Adjusted for age, serological status, smoking, glucocorticoid use at RA diagnosis, educational level, and calendar period. DAS28, 28-joint Disease Activity Score; RA, rheumatoid arthritis; RR, relative risk.

BMJ Publishing Group Limited (BMJ) disclaims all liability and responsibility arising from any reliance placed on this supplemental material which has been supplied by the author(s)

Supplemental table S17. Relative risk of failure to reach DAS28 remission at 3 and 6 months after RA diagnosis, by comorbidity categories and serological status

	**n (%) of seropositive patients with data on DAS28**	**Seropositive, n of observations 3m=3813 6m=2800 RR (95% CI)**	**n (%) of seronegative patients with data on DAS28**	**Seronegative, n of observations 3m=1601 6m=1130 RR (95% CI)**
Cardiovascular, 3m	396 (68.9)	0.93 (0.81, 1.08)	221 (65.8)	1.12 (0.92, 1.36)
Cardiovascular, 6m	235 (40.9)	0.99 (0.82, 1.20)	145 (43.2)	1.13 (0.89, 1.44)
Non-cardiac vascular, 3m	1330 (67.3)	1.03 (0.94, 1.12)	735 (67.2)	0.99 (0.87, 1.12)
Non-cardiac vascular, 6m	877 (44.4)	1.06 (0.94, 1.20)	509 (46.6)	1.01 (0.87, 1.18)
Malignant, 3m	200 (70.7)	0.91 (0.77, 1.08)	107 (64.1)	0.87 (0.71, 1.06)
Malignant, 6m	124 (43.8)	1.02 (0.80, 1.30)	79 (47.3)	1.07 (0.78, 1.46)
Endocrine, 3m	993 (65.9)	**1.12 (1.02, 1.24)**	461 (69.4)	0.94 (0.82, 1.08)
Endocrine, 6m	721 (47.9)	1.10 (0.97, 1.24)	333 (50.2)	1.09 (0.91, 1.30)
Gastrointestinal, 3m	310 (69.4)	1.10 (0.94, 1.29)	138 (64.2)	1.10 (0.86, 1.40)
Gastrointestinal, 6m	218 (48.8)	1.01 (0.83, 1.22)	110 (51.2)	1.07 (0.82, 1.41)
Infectious, 3m	262 (63.7)	**1.28 (1.04, 1.58)**	111 (60.3)	1.26 (0.94, 1.67)
Infectious, 6m	172 (41.8)	1.02 (0.81, 1.28)	87 (47.3)	1.23 (0.90, 1.70)
Chronic kidney, 3m	22 (59.5)	1.17 (0.66, 2.05)	15 (65.2)	1.50 (0.57, 3.94)
Chronic kidney, 6m	11 (29.7)	1.33 (0.51, 3.46)	9 (39.1)	1.04 (0.30, 3.66)
Neurological, 3m	332 (67.1)	1.10 (0.95, 1.28)	184 (71.3)	1.14 (0.92, 1.41)
Neurological, 6m	254 (51.3)	1.20 (0.99, 1.47)	133 (51.6)	**1.36 (1.02, 1.82)**
Psychiatric, 3m	750 (67.1)	**1.26 (1.12, 1.41)**	330 (66.4)	**1.23 (1.03, 1.47)**
Psychiatric, 6m	564 (50.4)	**1.33 (1.15, 1.55)**	254 (51.1)	1.17 (0.95, 1.44)
Respiratory, 3m	235 (65.5)	**1.24 (1.01, 1.52)**	103 (69.6)	1.13 (0.84, 1.52)
Respiratory, 6m	178 (49.6)	1.23 (0.96, 1.58)	74 (50.0)	1.05 (0.76, 1.45)

Adjusted for sex, age, smoking, glucocorticoid use at RA diagnosis, educational level, and calendar period. DAS28, 28-joint Disease Activity Score; RA, rheumatoid arthritis; RR, relative risk.
